# Prevalence of overweight and obesity among adolescents living with HIV after dolutegravir - based antiretroviral therapy start in Kampala, Uganda

**DOI:** 10.1186/s12981-024-00615-6

**Published:** 2024-04-18

**Authors:** Irene Nakatudde, Elizabeth Katana, Eva Laker Agnes Odongpiny, Esther Alice Nalugga, Barbara Castelnuovo, Mary Glenn Fowler, Philippa Musoke

**Affiliations:** 1 Infectious Diseases Institute, College of Health Sciences, Makerere University, Kampala, Uganda; 2 College of Health Sciences, Makerere University, Kampala, Uganda; 3 School of Medicine, University of St. Andrews, St. Andrews, Scotland; 4 Department of Pathology, School of Medicine, John Hopkins University, Baltimore, MD USA; 5 Makerere University-John Hopkins University Research Collaboration, Kampala, Uganda; 6 Department of Paediatrics and Child Health, College of Health Sciences, Makerere University, Kampala, Uganda

**Keywords:** Adolescents, HIV, Dolutegravir, Weight gain, Uganda

## Abstract

**Background:**

Dolutegravir (DTG)-based antiretroviral therapy (ART) is currently the preferred first-line treatment for persons living with HIV (PLHIV) including children and adolescents in many low- and middle-income countries including Uganda. However, there are concerns about excessive weight gain associated with DTG especially in adults. There remains paucity of current information on weight-related outcomes among adolescents on DTG. We determined the prevalence of excessive weight gain and associated factors among adolescents living with HIV (ALHIV) receiving DTG-based ART in Kampala, Uganda.

**Methods:**

Cross-sectional study involving ALHIV aged 10–19 years on DTG-based ART for at least one year were recruited from public health facilities in Kampala between February and May 2022. Excessive weight gain was defined as becoming overweight or obese per body mass index (BMI) norms while on DTG-based ART for at least one year. Demographic, clinical and laboratory data were collected using interviewer-administered questionnaires and data extracted from medical records. At enrolment, blood pressure and anthropometry were measured and blood was drawn for blood glucose and lipid profile. Data was summarised using descriptive statistics and logistic regression was performed to determine the associated factors.

**Results:**

We enrolled 165 ALHIV with a median age of 14 years (IQR 12–16). Eighty (48.5%) were female. The median duration on ART and DTG was 8 years (IQR 7–11) and 2 years (IQR 1–3) respectively. At DTG initiation, the majority of participants (152/165, 92.1%) were ART-experienced, and had normal BMI (160/165, 97%). Overall, 12/165 (7.3%) adolescents (95% CI: 4.2–12.4) had excessive weight gain. No factors were significantly associated with excessive weight gain after DTG start in ALHIV. However, all ALHIV with excessive weight gain were females.

**Conclusion:**

Our study found a prevalence of 7.3% of overweight and obesity in ALHIV after initiating DTG. We did not find any factor significantly associated with excessive weight gain in ALHIV on DTG. Nonetheless, we recommend ongoing routine monitoring of anthropometry and metabolic markers in ALHIV as DTG use increases globally, to determine the exact magnitude of excessive weight gain and to identify those at risk of becoming overweight or obese while taking the medication.

## Background

In 2018, the World Health Organisation (WHO) recommended dolutegravir (DTG) - based antiretroviral therapy (ART) as the preferred first- and second- line treatment for PLHIV including children and adolescents in sub-Saharan Africa (SSA) [1]. Dolutegravir is a second-generation integrase strand transfer inhibitor (INSTI) and a relatively new antiretroviral drug. The recommendation to use DTG was premised on its superior efficacy, tolerability, higher genetic barrier to develop HIV drug resistance, few drug-drug interactions compared to non-nucleoside reverse transcriptase inhibitors (NNRTI)- based ART, the once preferred first-line ART [1, 2]. Despite this favourable profile, emerging evidence from case reports, observational studies and clinical trials show statistically significant weight gain and even clinical obesity associated with INSTIs particularly DTG especially in adults compared to other ART drug classes [2–8]. The ADVANCE trial in South Africa, found significantly more weight gain with DTG-containing regimens than with the standard-care regimens (Efavirenz (EFV)-based). At 48 weeks, absolute weight gain and the proportion of people in whom obesity emerged during treatment was highest with tenofovir alafamide fumarate (TAF)/ emitricitabine (FTC)/DTG (6 kg, 14% new obesity) and tenofovir disopril fumarate (TDF)/FTC/DTG (3 kg, 7% new obesity) compared with TDF/FTC/EFV (1 kg, 6% new obesity) [5]. Similarly, the NAMSAL trial in Cameroon reported obesity occurring more in participants in the DTG group (12.3%) compared to the EFV400 group (5.4%) at 48 weeks of treatment [6].

Weight gain following initiation of ART is common, often representing effective viral suppression and a “return to health” phenomenon, as a result of reversal of the catabolic effects of HIV infection and HIV-related inflammation as observed in both adults and children [9, 10]. However, excessive weight gain, which places individuals in the obese or overweight category is undesirable and may contribute to the growing burden of non-communicable diseases (NCDs) such as cardiovascular disease and diabetes in PLHIV, who are already at higher risk of developing NCDs compared to people without HIV [10–12]. Factors associated with excessive weight gain while on DTG vary among studies [5, 8, 13, 14]. However, female sex, black race, low CD4 cell count, high pre-treatment HIV RNA load at baseline, being ART naïve, older age (≥ 60 years) and treatment with TAF as a companion drug to DTG have all been noted to be risk factors for greater weight gain with DTG use in the adult population [5, 8, 13–15].

With the ongoing roll out of DTG in the majority of SSA countries including Uganda, adolescents living with HIV (ALHIV) form a growing proportion of those receiving the drug. While studies have documented that DTG is safe and efficacious in ALHIV [16, 17], excessive weight gain associated with DTG is especially problematic for adolescents who are in a critical period of human growth and development. ALHIV experience multiple forms of stigma, undesired or unintended weight gain due to ART can affect their self-image/self-esteem, which may result in poor adherence and subsequent treatment failure [18]. Moreover, ALHIV are on life-long ART, and in resource-constrained settings where routine monitoring for metabolic markers such as blood glucose, lipids and blood pressure is rarely done, complications arising from excessive weight gain such as diabetes mellitus, dyslipidemia and hypertension may go unchecked.

To date, data on DTG- related weight gain among ALHIV is quite limited and available information is conflicting [19, 20]. Some studies have observed significant increase in BMI after switching from other regimens to DTG [21, 22], while others have not [23–25]. For example, no excessive weight gain was observed among 66 perinatally HIV-infected young adults and adolescents switched to INSTI compared to non-INSTI based regimens in a 10-year observational study in Italy [23]. While ODYSSEY, a randomised multi-country trial among children < 18 years from SSA, Thailand and Europe reported few (4%) children became overweight and obese while on DTG at 96 weeks of treatment [24]. In contrast, significant body mass index (BMI) increase was observed among a cohort of 460 adolescents with viral suppression transitioning to DTG at 1 year at an HIV treatment center in Eswatini [21].

In Uganda, transition to DTG among ALHIV began in 2018 as per the Ministry of Health guidelines [26]. In this study we determined the proportion of ALHIV who became overweight and obese at least one year following DTG start and the associated factors in an urban setting in Uganda.

## Methods

### Study design

This was a cross-sectional study in which we conducted a single visit that included a face-to-face interview, anthropometric measurements and laboratory investigations. In addition, previous clinical information was extracted from the participants’ files. In this study we determined the period prevalence of excessive weight gain in ALHIV after initiating DTG within the last 5 years.

### Study setting

The study was conducted at the Kampala Capital City Authority (KCCA) health centres in Kampala, the capital city of Uganda between February and May 2022. There are six health centres that are public health facilities supported by the Infectious Diseases Institute through the U.S President’s Emergency Plan for AIDS Relief to provide comprehensive HIV prevention, care and treatment services to PLHIV, including adolescents in the Kampala metropolitan area. Approximately, 1,300 adolescents are enrolled in care at these facilities and have a designated day for the adolescent clinic where they are provided with a range of services including; comprehensive HIV prevention, care and treatment, sexually transmitted infections management, family planning and counselling. Clinic visits are scheduled every 3–6 months, and on these visits, anthropometric measurements are taken including, weight and height. Viral load measurements are done every six months from the National Public Health Laboratory as per the Ministry of Health (MOH) guidelines and CD4 cell count measurement is no longer recommended. Adherence to ART at each visit is documented. Following the release of the consolidated guidelines for the prevention and treatment of HIV and AIDS in Uganda by the MOH in 2020, there is an on-going nationwide DTG rollout in all health facilities among PLHIV including adolescents with recommendation to initiate eligible treatment naïve patients to DTG-based ART as well as switch treatment experienced patients with virologic suppression (defined as < 1,000 copies/ml within the last 6 months) from EFV or protease inhibitor (PI)-based regimens to DTG. Abacavir, Zidovudine, Lamivudine, and TDF are the recommended companion drugs to DTG in our setting [27].

### Study population

We included 165 ALHIV aged 10–19 years on DTG-based ART for at least one year attending the ART clinic in KCCA health centres. Adolescents 18 years or older and emancipated minors provided written informed consent. Written parental consent and assent was obtained for those aged 10–17 years who were not emancipated minors. Adolescents with missing baseline height and weight at DTG initiation, unknown DTG start date, those on antipsychotic medications, steroids or with underlying chronic medical conditions such as diabetes mellitus, renal, thyroid and cardiac disease and those that were pregnant while on DTG-based ART were excluded. In addition, ALHIV who declined to participate, were unreachable when contacted or had date of birth discrepancies were excluded from the study.

### Sample size

The sample size of 165 was estimated using the Modified Kish Leslie formula for cross-sectional studies, based on the prevalence of overweight in youth living with perinatal HIV infection on antiretroviral treatment in Thailand (11%) [28] and 10% non-response (missing information).

### Data collection procedures

Participants were recruited from three public health facilities that routinely collect both the weight and height anthropometric measurements during clinic visits. A list of ALHIV aged 10–19 years who were on DTG for at least one year was obtained from each of these health facilities. We consecutively reviewed and screened clinic files of ALHIV to identify those eligible for inclusion in the study. Eligible participants and/or parents/caretakers where contacted via phone and invited to participate in the study at a convenient date. Participants were encouraged to fast (have last taken a meal, food or drink except for water at least 8 h prior) on the day of the study visit. Prior to enrolment, the research assistants provided details about the study to the participants and their parents/caretakers and eligibility was reassessed. Written informed consent, parental consent and assent were obtained accordingly. A structured questionnaire was used to collect socio-demographic and clinical data. We obtained information about pre-existing medical conditions, history of side effects after DTG start, smoking, alcohol intake and other substance use, concomitant medications, hormonal contraceptive use, family history of diabetes mellitus and hypertension and self report of involvement in physical activities that included exercise and sports such as running, aerobics, skipping ropes, football, etc. with in the past one month. In addition, we extracted data from the participant’s clinic file that included, date of ART and DTG-based regimen start, previous opportunistic infections, ART regimens, weight and height measurements and viral loads. We used viral load as a surrogate marker for medication adherence.

During the enrolment, anthropometric measurements including weight (kg) and height (cm) were taken as per WHO guidelines [29]. Weight was measured to the nearest 0.1 kg and height to the nearest millimetre with participants wearing light clothing and no shoes using a well-calibrated scale and stadiometer (Seca, Germany). BMI was defined as the weight in kilograms divided by the square of the height in meters (kg/m^2^) and classified according to WHO BMI-for-age z scores (5–19 years) as underweight (z-score <-2SD), normal weight (z-score ≥-2 and ≤ + 1SD), overweight (z-score > + 1SD (equivalent to BMI 25 kg/m^2^ at 19 years) and obesity (z-score > + 2SD (equivalent to BMI ≥ 30 kg/m^2^ at 19 years) [30]. A single blood pressure measurement was undertaken for each participant at rest using a calibrated OMRON M2 automatic upper arm blood pressure monitor and was graded as normal blood pressure < 120/80 mmHg and elevated blood pressure ≥ 120/80 mmHg for adolescents 13 years and older and normal if < 90th percentile and elevated if ≥ 90th percentile based on age, sex and height for adolescents 10–12 years [31].

Laboratory investigations: Fasting blood glucose (FBG) was measured using a calibrated On Call Plus blood glucose meter. FBG < 5.6 mmol/L was normal, pre-diabetic 5.6–6.9 mmol/L and high/diabetic if ≥ 7.0 or random blood glucose (RBG) > 11.1 mmol/L [32, 33]. Lipid profile was done for all participants and glycated haemoglobin (HbA1c) was done only for those with FBG ≥ 5.6 mmol/L. For HbA1c levels, a venous blood sample of 3 mL was drawn into an ethylenediamine tetraacetic acid blood collection tube from the study participant. The blood was quantitatively tested for HbA1c levels using the Roche COBAS Integra 400 plus automated analyser. HbA1c was categorised normal ≤ 5.6%, pre-diabetes 5.7–6.4% and diabetes ≥ 6.5% [33]. Similarly, for lipid profile, a venous blood sample of 4 mL was drawn into a plain (Red top) blood collection tube from each study participant. Blood was centrifuged at 3000 revolutions per minute (rpm) for ten minutes, and serum was collected in a Sarstedt 2 mL cryovial and quantitatively tested for lipid profile tests using the Roche COBAS Integra 400 plus automated analyser and categorised as follows; Triglycerides - acceptable < 90 mg/dl, borderline 90–129 mg/dl, high ≥ 130 mg/dl; Low- density lipoprotein cholesterol acceptable < 110 mg/dl, borderline 110–129 mg/dl, high ≥ 130 mg/dl; High-density lipoprotein cholesterol acceptable > 45 mg/dl, borderline 40–45 mg/dl, Low < 40 mg/dl; Total cholesterol acceptable < 170 mg/dl, borderline 170–199 mg/dl, high ≥ 200 mg/dl [34].

### Data management and statistical analysis

Data was analysed using STATA 14.0 (Stata Corp., College Station, TX, USA). Continuous variables were summarized with means and standard deviations for normally distributed data and with medians and interquartile ranges for data that is not normally distributed. Categorical variables were described using frequencies and, percentages.BMI and BMI-for-age z scores were calculated using WHO Anthro software [35]. . The primary outcome variable excessive weight gain was defined as an ALHIV who becomes overweight or obese at least one year after DTG initiation. Overweight or obesity were treated as binary outcomes. Chi-square or Fisher’s exact tests were used to compare participant characteristics between BMI categories (normal weight vs. overweight and obesity). Logistic regression was used to identify the factors associated with excessive weight gainafter DTG start. Bivariate analysis assessed the crude odds ratios of exposure variables. Variables included in the analysis were sex, ART experience, pre-switch anchor drug, current viral load, and duration of DTG treatment, lipid profile, blood pressure and glucose. Stepwise multiple logistic regression models were employed to control for confounding and interaction effects. Significant variables at the bivariate level (*p* ≤ 0.20) and those with clinical significance from literature were included in the multivariate model. Variables with a p-value of < 0.05 (two-sided) were considered statistically significant. Missing data were assessed and imputed using standard techniques.

## Results

### Study recruitment

**Fig. 1:**
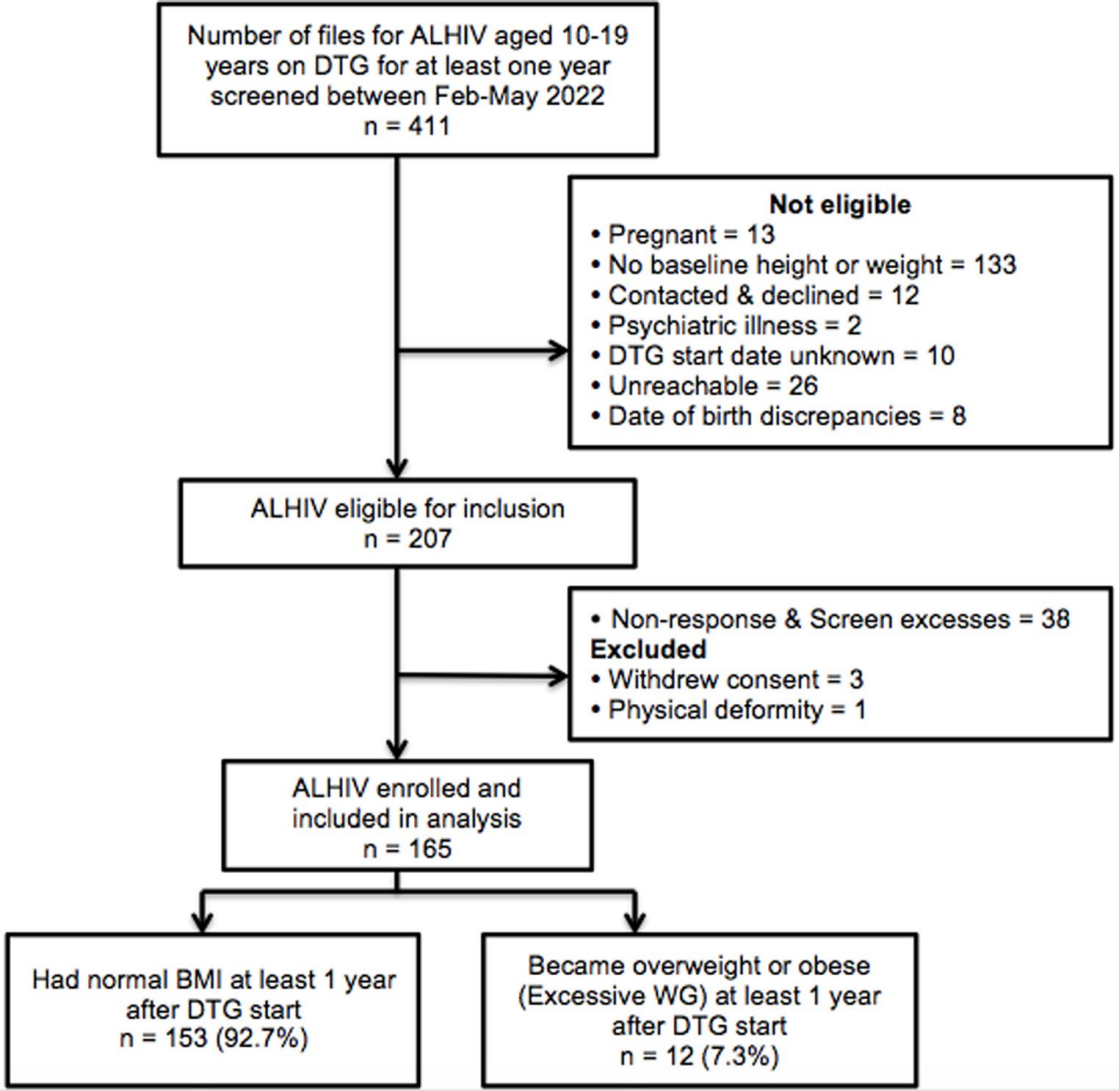
Study flow diagram

We reviewed 411 clinic files of ALHIV aged 10–19 years on DTG for at least one year between February and May 2022. Of these, 204 were not eligible for inclusion in the study. In addition, .three ALHIV were excluded because they withdrew their consent prior enrolment and one ALHIV was also excluded due to a limb deformity that impeded accurate measurement of anthropometry (height). We enrolled 165 eligible participants into the study, who were all included in the analysis (Fig. 1).

### Characteristics of the study participants

Of the 165 enrolled ALHIV, 80 (48.5%) were female; median age was 14.0 years (IQR 12–16). the median duration on ART and DTG was 8 years (IQR 7,11) and 2 years (IQR 1,3) respectively. At the time of DTG initiation, the majority of participants were ART- experienced 152/165 (92.1%), and 145/165 (87.9%) had achieved viral suppression at the most recent viral load. ALHIV that reported involvement in a physical activity were 135/165 (66.7%), 109/152 (71.7%) were on a NNRTI-based regimen and 43/152 (23.3%) were on a PI-based regimen prior DTG initiation. At DTG start, the majority of ALHIV had normal BMI, 160/165 (97%) while 5/165 (3%) were overweight and obese. At enrolment, 150/165 ALHIV (90.9%) had normal BMI while 15/165 (90.9%) were obese and overweight. There was no ALHIV who was underweight at DTG start or at enrolment. Overall 12/165 (7.3%) adolescents (95% CI: 4.2–12.4) became overweight or obese at least one year following DTG initiation. Of these, 2 were obese and 10 were overweight. All ALHIV with excessive weight gain were female, ART- experienced, had achieved viral suppression at the most recent viral load and had normal blood glucose. Table 1 shows additional clinical and laboratory characteristics of the participants by primary outcome.

**Table 1:** Clinical and laboratory characteristics of ALHIV on DTG by current BMI in Kampala, Uganda

Characteristic	Normal BMI n = 153 (%)	Overweight/Obese (Excessive weight gain) n = 12 (%)	Total N = 165 (%)	P-value
**Sex**				0.001
Female	68 (44.4)	12 (100)	80 (48.5)	
Male	85 (56.6)	0	85 (51.5)	
**Age (years)**				0.357
10–14	88 (57.5)	8 (66.7)	96 (58.2)	
15–17	45 (29.4)	4 (33.3)	49 (29.7)	
18–19	20 (13.1)	0 (0)	20 (12.1)	
**ART experience prior DTG start**				0.601
ART- experienced	140 (91.5)	12 (100)	152 (92.1)	
ART- naïve	13 (8.5)	0 (0)	13 (7.9)	
**Duration on DTG (years)**				
1 - < 2	39 (25.5)	4 (33.3)	43 (26.1)	0.862
2 - < 3	46 (30.1)	3 (25.0)	49 (29.7)	
3 - 4	68 (44.4)	5 (41.7)	73 (44.2)	
**Reported history of side effects^ after DTG start**				0.001
No	130 (85.0)	7 (58.3)	137 (83.0)	
Yes	23 (15.0)	5 (41.7)	28 (17.0)	
**Viral load at DTG start**				0.731
Suppressed (< 1000 copies/ml)	133 (86.9)	12 (100)	145 (87.9)	
Unsuppressed (≥ 1000 copies/ml)	5 (3.3)	0 (0)	5 (3.0)	
Unknown/Not applicable^¶^	15 (9.8)	0 (0)	15 (9.1)	
**Current viral load**				0.609
Suppressed (< 1000 copies/ml)	142 (92.8)	11 (91.7)	153 (92.7)	
Unsuppressed (≥ 1000 copies/ml)	5 (3.3)	0 (0)	5 (3.2)	
Unknown (Missing data)	6 (3.9)	1 (8.3)	7 (4.2)	
**Involvement in physical activity**				0.691
No	26 (17.0)	4 (33.3)	30 (18.2)	
Yes	127 (83.0)	8 (66.7)	135 (81.8)	
**Family history of hypertension or diabetes mellitus**				0.445
No	79 (51.6)	5 (41.7)	84 (50.9)	
Yes	61 (39.8)	7 (58.3)	68 (41.2)	
Unknown	13 (8.5)	0 (0)	13 (7.9)	
**Smoking, alcohol and substance use**				0.939
No	153 (100)	11 (91.7)	164 (99.4)	
Yes	0 (0)	1 (8.3)	1 (0.6)	
**Blood pressure**				0.164
Normal (< 90th percentile /< 120/80 mmHg)	90 (58.8)	8 (66.7)	98 (59.4)	
Elevated (≥ 90th percentile / ≥ 120/80 mmHg)	63 (41.2)	4 (33.3)	67 (40.6)	
**Blood glucose**				0.570
Normal (FBG < 5.6mmol/L or RBG < 11.1mmol/L)	141 (92.2)	12 (100)	153 (92.7)	
Pre-diabetic (FBG 5.6–6.9 mmol/L)	12 (7.8)	0 (0)	12 (7.3)	
Diabetic (FBG ≥ 7.0 mmol/L)	0 (0)	0 (0)	0 (0)	
**Triglycerides**				0.887
Acceptable (< 90 mg/dl)	90 (58.8)	9 (75.0)	99 (60)	
Borderline (90–129 mg/dl)	47 (30.7)	3 (25.0)	50 (30.3)	
High (≥ 130 mg/dl)	16 (10.5)	0 (0)	16 (9.7)	
**Total cholesterol**				0.081
Acceptable (< 170 mg/dl)	141 (92.2)	10 (83.3)	151 (91.5)	
Borderline (170–199 mg/dl)	12 (7.8)	1 (8.3)	13 (7.9)	
High (≥ 200 mg/dl)	0 (0)	1 (8.3)	1 (0.6)	
Low-density lipoprotein **cholesterol**				0.180
Acceptable (< 110 mg/dl)	142 (92.8)	10 (83.3)	152 (92.1)	
Borderline (110–129 mg/dl)	11 (7.2)	2 (16.7)	13 (7.9)	
High (≥ 130 mg/dl)	0 (0)	0 (0)	0 (0)	
**High-density lipoprotein cholesterol**				0.093
Acceptable (> 45 mg/dl)	30 (19.6)	4 (33.3)	34 (20.6)	
Borderline (40–45 mg/dl)	33 (21.6)	3 (25.0)	36 (21.8)	
Low (< 40 mg/dl)	90 (58.8)	5 (41.7)	95 (57.6)	

**^** Side effects reported included; Abdominal pain, Blurred vision, Dizziness, General body weakness, Headache, Hives, Increased appetite, Nausea, Oral sores, Vomiting, Weight gain

^¶^Viral load test not a requirement for ART-naïve patients prior ART start

Bivariate and multivariate analysis of factors associated with excessive weight gain in ALHIV after starting DTG yielded non-significant results, suggesting that the variables examined may not have a statistically significant association with excessive weight gain (Table 2). Variables analysed included; sex, ART-experience, pre-switch anchor ARV drugs, duration of DTG treatment, involvement in physical activities, blood pressure, most recent viral load, blood glucose, and lipid profile (Table 2).

**Table 2:** Bivariate and multivariate analysis of factors associated with excessive weight gain in ALHIV on DTG in Kampala, Uganda

Characteristic	Normal Weight n = 153 (%)	Excessive weight gain n = 12 (%)	Crude OR (95%CI)	P-value	Adjusted OR (95%CI)	P-value
**Sex**						
Male	85 (55.6)	0	1.00			
Female	68 (44.4)	12 (100)	∞			
**ART experience**						
Treatment-naïve prior DTG start	13 (8.5)	0	1.00 (ref)			
Treatment-experienced prior DTG start	140 (91.5)	12 (100)	∞			
**NRTI backbone prior DTG start**						
Abacavir-based	57 (40.7)	4 (33.3)	1.00			
Zidovudine-based	63 (45.0)	7 (58.3	1.58 (0.44–5.69)	0.482		
TDF-based	20 (14.3)	1 (8.3)	0.71 (0.08–6.76)	0.768		
NNRTI backbone* prior DTG start						
EFV/NVP-based	101(72.1)	8 (66.7)	1.00 (ref)			
PI-based	39 (27.9)	4 (33.7)	1.29 (0.37–4.55)	0.687		
**Duration on DTG**						
1-<2yr	39 (25.5)	4 (33.3)	1.00 (ref)		1.00 (ref)	
2-<3yr	46 (30.1)	3 (25.0)	0.64 (0.13–3.02)	0.569	0.60 (0.12–2.96)	0.380
3- <5yr	68 (44.4)	5 (41.7)	0.73 (0.18–2.83)	0.635	0.88 (0.21–3.69)	0.939
**Viral load at DTG start**						
Unsuppressed (≥ 1,000 copies/ml)	5 (3.3)	0	1.00 (ref)			
Suppressed (< 1,000 copies/ml)	133 (86.9)	12 (100)	∞			
Unknown/Not applicable	15 (9.8)	0	∞			
**Current viral load**						
Unsuppressed (≥ 1,000 copies/ml)	5 (3.3)	0	∞			
Suppressed (< 1,000copies/ml)	142 (92.8)	11 (91.7)	0.46(0.05–4.21)	0.496	041(0.04–4.24)	0.453
Unknown/Not applicable	6 (3.9)	1 (8.3)	∞		∞	
**Experienced drug side effects after DTG start**						
No	130 (85.0)	7 (58.3)	1.00 (ref)		1.00 (ref)	
Yes	23 (15.0)	5 (41.7)	4.04 (1.18–13.8)	0.026	4.20 (1.20-14.71)	0.025
**Physical activity**						
No	26 (17.0)	4 (33.3)	1.00 (ref)			
Yes	127 (83.0)	8 (66.7)	0.41 (0.11–1.46)	0.169		
**Previous opportunistic infections**						
No	147 (96.1)	11 (91.7)	1.00 (ref)			
Yes	6 (3.9)	1 (8.3)	2.23 (0.25–20.2)	0.476		
**Blood pressure**						
Normal (< 90th percentile /<120/80 mmHg)	90 (58.8)	8 (66.7)	1.00 (ref			
Elevated (≥ 90th percentile /≥120/80mmHg)	63 (41.2)	4 (33.3)	0.714 (0.21–2.46)	0.596		
**Blood glucose**						
Normal	141 (92.2)	12 (100)	1.00 (ref)			
Pre-diabetic	12 (7.8)	0	∞			
High	0	0	∞			
**Triglycerides**						
Acceptable (< 90 mg/dl)	90 (58.8)	9 (75.0)	1.00 (ref)			
Borderline High (90-129 mg/dl)	47 (30.5)	3 (25.0)	0.64 (0.16–2.87)	0.516		
High (≥ 130 mg/dl)	16 (10.5)	0	∞			
**Total cholesterol**						
Acceptable (< 170 mg/dl)	141 (92.2)	10 (83.3)	1.00 (ref)			
Borderline High (170-199 mg/dl)	12 (7.8)	1 (8.3)	1.18 (0.14–9.94)	0.882		
High (≥ 200 mg/dl)	0	1 (8.3)	∞			
**Low density lipoprotein cholesterol**						
Acceptable (< 110 mg/dl)	142 (92.8)	10 (83.3)	1.00 (ref)			
Borderline High (110-129 mg/dl)	11 (7.2)	2 (16.7)	2.58 (0.50–13.2)	0.256		
High (≥ 130 mg/dl)	0	0	∞			
**High density lipoprotein cholesterol**						
Acceptable (> 45 mg/dl)	30 (19.6)	4 (33.3)	1.00 (ref)			
Borderline Low (40-45 mg/dl)	33 (21.6)	3 (25.0)	0.68 (0.14–3.29)	0.634		
Low (< 40 mg/dl)	90(58.8)	5 (41.7)	0.42 (0.11–1.65)	0.213		

*N = 152

## Discussion

Currently, the WHO recommends DTG - based ART as the preferred first- and second- line treatment for ALHIV. In this study, we found that 7.3% (12/165) of ALHIV had excessive weight gain at least one year after the start of a DTG-based regimen. To date, literature on DTG-related weight gain and its prevalence in ALHIV remains limited globally, but recently, ODYSSEY, an open-label randomised clinical trial found a prevalence of 4% of new overweight and obesity among children < 18 years majorly from SSA on DTG at 96 weeks of treatment [24]. In addition, significant BMI increase was observed among a cohort of 460 adolescents with viral suppression transitioning to DTG at 1 year in Eswatini [21]. Similarly, a retrospective study of 38 children and youth aged 0–19 years in the United States found a high BMI change after initiating DTG, the median follow-up time of 527 days [20]. Findings from these studies and ours show that DTG-related weight gain is prevalent in ALHIV; however, other factors that contribute to weight gain should also be considered, as the causes are often multifactorial.

Our study did not find factors significantly associated with excessive weight gain and DTG in ALHIV. Furthermore, factors previously documented to be associated with excessive weight after DTG start among adults such as female sex, high pre-treatment viral load and previous ART anchor drug [14, 15] were also not significant in our study. This could be due to the difference in the characteristics of the populations or the small size of participants for each of these factors or the lack thereof limiting our ability to analyse these associations robustly in our study. However, our study found that all ALHIV with excessive weight gain were female. This observation is not new; as it has previously been documented in multiple studies on DTG and weight gain in adults [5, 14, 15]. Similarly, female sex has also been documented as a modifying factor between DTG and rate of weight gain or BMI change in adolescents. A retrospective observational cohort study among 460 virally supressed ALHIV in Eswatini reported the rate of BMI change after transition to DTG was increased by 1.1 kg/m^2^ in females compared to 0.6 kg/m^2^ in males per year [21]. However, the underlying mechanisms for higher weight gain among females as opposed to males remain unknown [15].

Key metabolic markers such as blood pressure, blood glucose and lipid profile and physical activity were explored in this study. However, we did not find significant association between excessive weight gain after DTG start and blood pressure, blood glucose or dyslipidemia and physical activity at bivariate analysis. For example, all the participants with excessive weight gain after DTG start in our study had normal blood glucose levels. This is contrary to what was observed in a study among adults living with HIV on DTG in Uganda of increased risk of hyperglycaemia among those with obesity and overweight compared to those with normal BMI after initiating DTG [36]. There remain contrasting observations between adolescents and adults with regard to hyperglycemia and BMI changes while on DTG and the reasons for this are still unclear. Our study may not have observed any significant association between metabolic markers and excessive weight gain, however, on-going monitoring of metabolic markers and the impact of DTG on weight gain in ALHIV should be done as its use becomes widespread in this population.

### Study strengths and limitations

To our knowledge, this is the first study in Uganda documenting the proportion of ALHIV who became overweight and obese after transitioning to DTG-based ART. Our study contributes to the body of evidence addressing one of the key gaps in data on weight gain and new ARV drugs highlighted by the WHO in its updates on the transition to DTG-based ART, 2022. However, our study is limited by the fact that we used retrospective data; routine clinical data especially in resource-limited settings may not be rigorously collected and this was compounded by the recent COVID-19 lockdowns. Hence, anthropometric data was not available for all time points and all participants, with some potential participants ineligible for inclusion in the study due to missing weight and height at DTG start. Therefore, there may have been selection and information bias, affecting the generalizability of our findings to other settings/populations. Moreover, the study was carried out mainly in an urban population, which may differ significantly to ALHIV in rural settings. Also there is a potential for recall bias in the reporting of past events. We were able to obtain and include information on other factors such as blood glucose, physical activity, smoking, alcohol and substance use that could affect weight gain. However, although we obtained data on physical activity, we acknowledge not using a standardised tool as a limitation. Our study also did not have a comparison group such as ALHIV on non-DTG based regimens as the majority of ALHIV in Uganda have now been transitioned to DTG as per MOH recommendations. Nonetheless, our study provides important new preliminary findings in this unexplored research area, among a unique population and has the potential to guide future research.

## Conclusion

Our study found a prevalence of 7.3% of overweight and obesity in ALHIV after initiating DTG. Regarding prognostic factors, our study did not find associations between excessive weight gain and factors previously documented in adults living with HIV such as female sex, high pre-treatment viral and the type of the pre-switch anchor drug. Nonetheless, we recommend ongoing routine monitoring of anthropometry and metabolic markers in ALHIV on DTG to determine the exact magnitude of excessive weight gain as DTG use increases globally in this population. In addition, more research using larger sample sizes, different study designs and carried out in various and different settings is required to identify ALHIV at risk of becoming overweight and obese while on DTG.

## Data Availability

The datasets used and/or analysed during the current study are available from the corresponding author on reasonable request.

## Abbreviations

AIDS: Acquired Immune Deficiency Syndrome
ALHIV: Adolescents Living with HIV
ART: Antiretroviral therapy
BMI: Body Mass Index
DTG: Dolutegravir
EFV: Efavirenz
FBG: Fasting blood glucose
HIV: Human Immunodeficiency Virus
INSTI: Integrase Strand Transfer Inhibitor
KCCA: Kampala Capital City Authority
MOH: Ministry of Health
NCDs: Non-Communicable Diseases
NRTI: Nucleotide Reverse Transcriptase Inhibitors
NNRTI: Non-Nucleotide Reverse Transcriptase Inhibitors
PI: Protease Inhibitor
PLHIV: Persons Living With HIV
RBG: Random blood glucose
SSA: Sub-Saharan Africa
TAF: Tenofivir Alafenamide Fumarate
TDF: Tenofovir Disoproxil Fumarate
WHO: World Health Organization
